# A case of multiple solitary neurofibromas located in the hypopharynx and cervical esophagus

**DOI:** 10.1002/deo2.160

**Published:** 2022-08-24

**Authors:** Hamada Kazu, Shimode Yuzo, Hata Yoshiyuki, Kunou Hiroaki, Kobayashi Rika, Matsunaga Kazuhiro, Kawaura Ken, Mukai Tsuyoshi, Kitakata Hidekazu, Itoh Tohru, Okuro Masashi, Yamada Sohsuke

**Affiliations:** ^1^ Department of Gastroenterological Endoscopy Kanazawa Medical University Ishikawa Japan; ^2^ Department of Geriatric Medicine Kanazawa Medical University Ishikawa Japan; ^3^ Department of Head and Neck Surgery Kanazawa Medical University Ishikawa Japan; ^4^ Department of Pathology and Laboratory Medicine Kanazawa Medical University Ishikawa Japan

**Keywords:** biopsy, endoscopy, esophagus, hypopharynx, neurofibroma

## Abstract

A 50‐year‐old female was admitted to our hospital because of mild throat discomfort. Esophagogastroduodenoscopy demonstrated multiple 2–3 mm diameter whitish granules in her hypopharynx and cervical esophagus. Based on biopsy specimens, we considered the lesions to represent normal tissue or very mild mucosal dysplasia. However, S‐100 immunohistochemical staining 3 years later led to a diagnosis of multiple solitary neurofibromas located in the hypopharynx and cervical esophagus. Histological finding characteristics of neurofibroma were present in 72.7% of hypopharyngeal biopsy specimens and 33.3% of esophageal specimens. When diagnosing neurofibroma, it is important to perform biopsies at multiple locations and at different times and to enlist the cooperation of endoscopists and pathologists for immunohistochemical staining with S‐100.

## INTRODUCTION

Neurofibroma is a benign peripheral nerve tumor that is derived from Schwann cells, perineural cells, and fibroblasts.^1^ While single neurofibromas located in the pharyngolarynx or esophagus have been reported, multiple neurofibromas in these locations are extremely rare. Here, we report the case of multiple solitary neurofibromas located in the hypopharynx and cervical esophagus.

## CASE REPORT

A 50‐year‐old female was admitted to our hospital because of mild throat discomfort. An otolaryngologist identified whitish granules in the hypopharynx and requested that a gastroenterological endoscopist assess the hypopharynx and upper digestive tract using esophagogastroduodenoscopy (EGD). To increase the likelihood of detecting any lesions that were present, we performed sedation with diazepam and pentazocine and attached a transparent cylinder to the endoscope.

**FIGURE 1:**
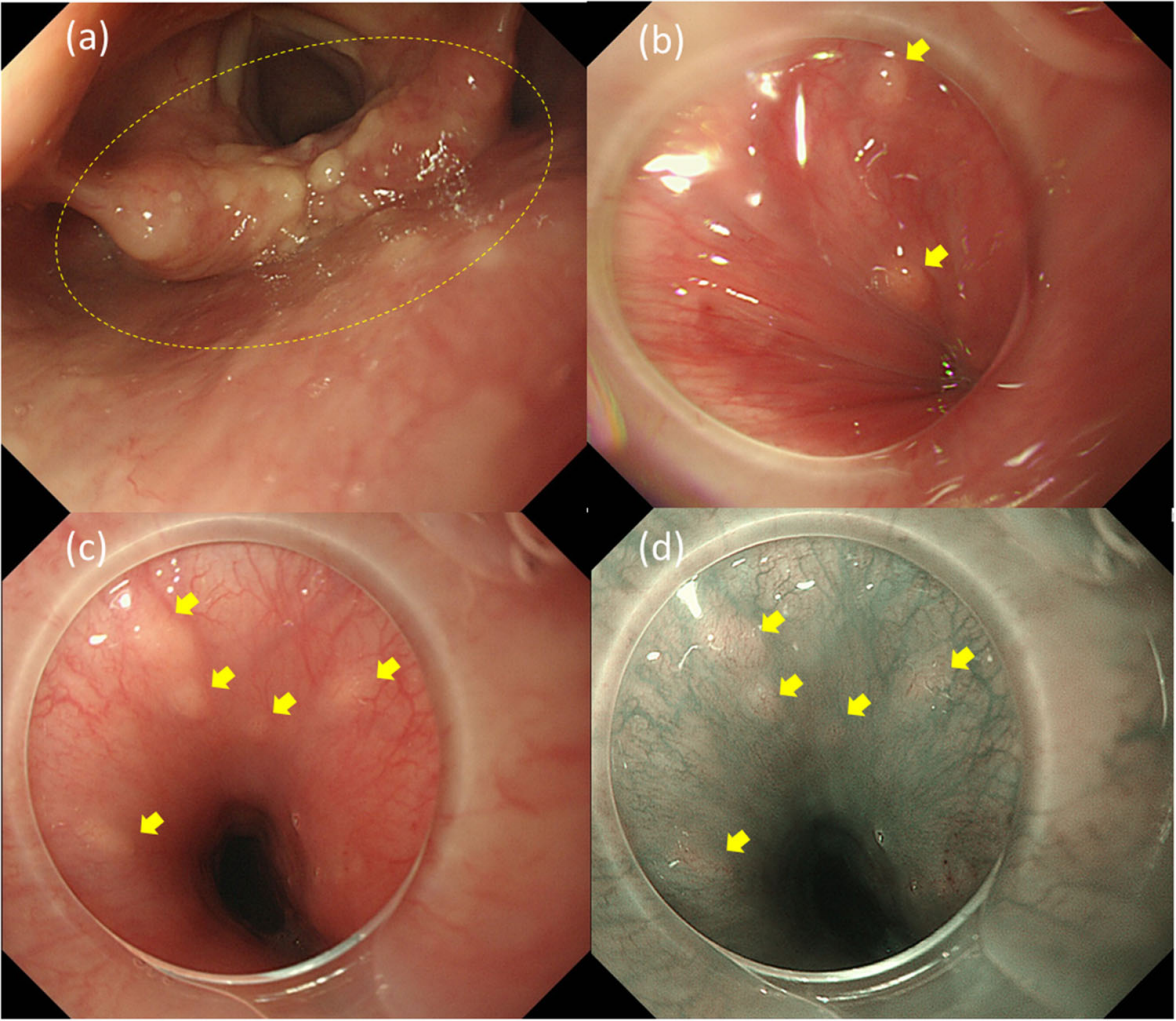
Endoscopic findings of the hypopharynx and cervical esophagus at the first examination. There are multiple whitish granules from the arytenoid to the cervical esophagus (yellow circle, yellow arrows): (a) laryngopharynx (ordinary white light), (b) left hypopharynx (ordinary white light), (c) cervical esophagus (ordinary white light), and (d) cervical esophagus (narrow band imaging)

**FIGURE 2:**
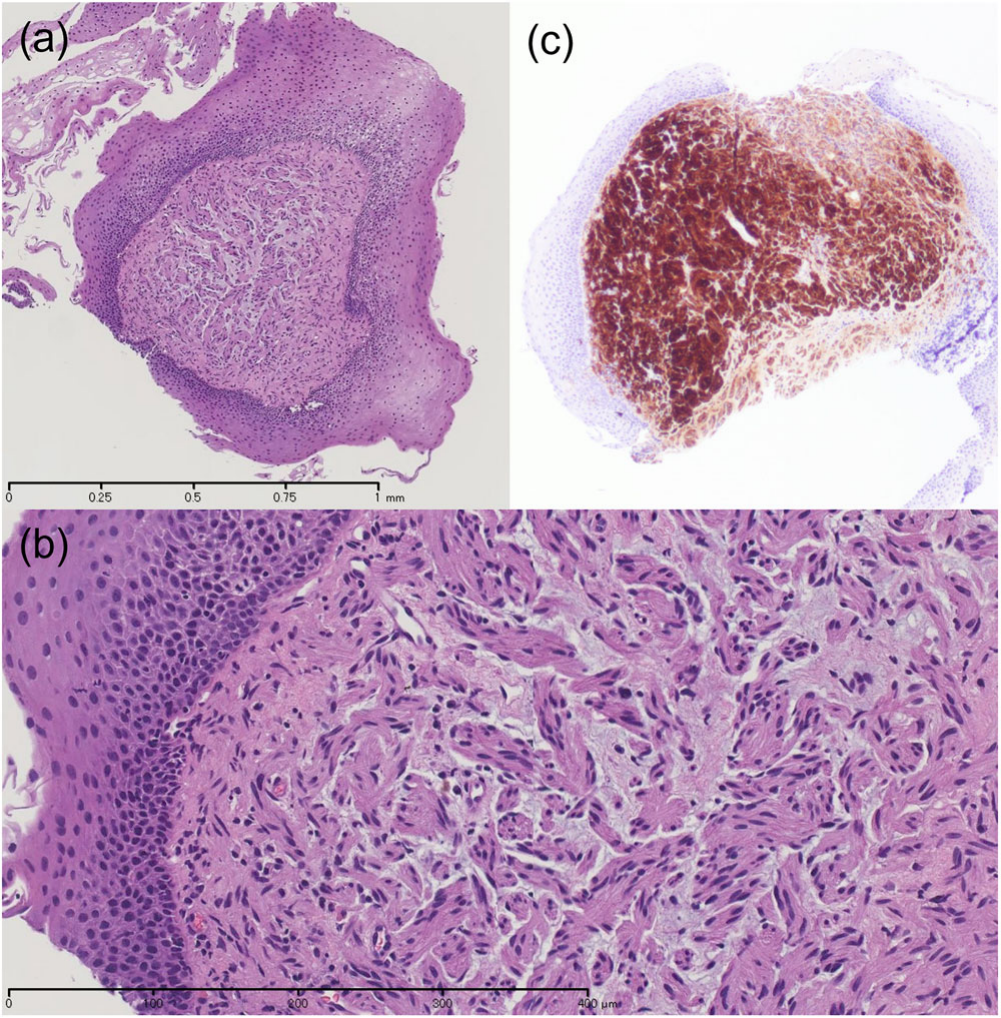
Pathological findings of an endoscopic biopsy specimen obtained from a hypopharyngeal lesion 3 years after the first examination. Submucosal tumors without capsule formation are composed of spindle cells under hematoxylin–eosin (HE) staining, and they are positive for immunohistochemical staining with S‐100. (a) HE staining: ×40, (b) HE staining: ×100, and (c) S‐100 staining: ×40

EGD demonstrated multiple 2–3 mm diameter whitish granules in the hypopharynx and cervical esophagus, approximately 13–16 cm from the incisor teeth (Figure 1). The lesions in the hypopharynx were slightly larger and denser than those in the esophagus. Based on biopsy specimens at that time, we considered the lesions to represent normal tissue or very mild mucosal dysplasia. We discussed observational follow‐up or microsurgery with the patient, and she chose follow‐up, with EGD and biopsy once a year. Three years after the initial examination, biopsy specimens obtained by EGD revealed submucosal tumors without capsular formation. They comprised spindle cells with positive immunohistochemical staining for S‐100 (Figure 2), and these pathological characteristics were consistent with a diagnosis of neurofibroma. When re‐evaluating past biopsy specimens, we found the same pathological findings in some of the lesions. The patient had no other factors that were suggestive of neurofibromatosis (NF), including a family history of NF, subcutaneous masses, and café au‐lait spots. We diagnosed multiple solitary neurofibromas located in the hypopharynx and cervical esophagus.

At the time of this writing, 8 years after the initial diagnosis, the lesions have not changed. We collected a total of 29 biopsy specimens derived by EGD at various points in time and detected neurofibroma in 72.7% (8/11) of the hypopharyngeal specimens and 33.3% (6/18) of the esophageal specimens.

## DISCUSSION

Neurofibroma may be classified as part of NF or as a solitary lesion that occurs sporadically.^2^ Most laryngeal neurofibromas are associated with NF,^3^ but most esophageal neurofibromas are solitary.^4^ The mean age of the patients with esophageal solitary neurofibromas at the time of diagnosis was 52.7 years (range: 26–75 years), and there was no difference between the sexes. Lesions were located primarily in the mid to upper section of the esophagus.^4^ Benign esophageal tumors were found in 0.17% of autopsies,^5^ and esophageal neurofibroma comprised only 1% of these tumors.^6^ Most of gastrointestinal solitary neurofibromas existed in the stomach, and esophageal solitary neurofibromas accounted for approximately 10% of them.^7^ Thus, neurofibromas located in the esophagus are highly unusual. Furthermore, most neurofibromas located in the pharyngolarynx or esophagus are single, large lesions with a diameter over 10 mm.^4^, ^8^ Multiple solitary neurofibromas that have diameters of only a few millimeters and present in highly circumscribed regions are extremely rare.^9^ We identified only one previously described case by Ishii et al., in which multiple solitary neurofibromas were located in the pharyngolarynx and esophagus, as in our patient.^10^ In that report, the neurofibromas were thought to originate from the superior laryngeal nerve,^10^ which was also probably the case in our patient because of the similar lesion distribution.

Small lesions in the cervical esophagus may often be overlooked due to factors such as lumen bending and natural constriction. We identified the neurofibroma lesions in the cervical esophagus in this case because we performed sedation and attached a transparent hood on the endoscope to specifically evaluate the existence of esophageal lesions. However, it could be challenging to identify such lesions without these preparations. They are particularly important when the detection of neurofibromas in the cervical esophagus might be difficult due to factors such as hypopharyngeal findings and NF.

The majority of neurofibromas in the pharyngolarynx and esophagus are benign. It is considered appropriate to perform enucleation for neurofibromas with symptoms and perform observational follow‐up for asymptomatic cases. While it is preferable to diagnose neurofibromas by biopsy to avoid excessive surgery, diagnosis with biopsy or fine needle aspiration is sometimes difficult.^4^, ^10^ There are no reports on the sensitivity of biopsies for the solitary neurofibromas in the hypopharynx and esophagus. In this case, such findings were detected in 72.7% of hypopharyngeal biopsied specimens and 33.3% of esophageal biopsied specimens. There are several potential reasons why the rate was lower in the esophagus. First, the hypopharyngeal lesions were slightly bigger than the esophageal lesions. Second, the muscularis mucosae in the esophagus might make it difficult to collect tumors by biopsy because esophageal neurofibromas originate in the submucosa or muscular layer,^8^ and the hypopharynx does not have muscularis mucosae. Third, factors related to endoscopic biopsy may have been involved; for instance, hypopharyngeal lesions can be identified and specimens collected using a vertical approach, but esophageal lesions can be accessed endoscopically only from a tangential direction. In any case, the sensitivity of diagnosing a neurofibroma based on only one biopsy sample is not very high, but the pathological diagnosis should be possible following multiple biopsies. Endoscopists should suspect neurofibromas when there are multiple whitish granules in the pharynx and esophagus, and biopsies should be performed at several locations and/or times for pathological examination. Maybe contrivances such as using big forceps may be useful for the improvement of the diagnosis.

Immunohistochemical staining using S‐100 is also useful for diagnosing neurofibromas,^1^, ^4^ but hematoxylin–eosin staining alone is usually ineffective. Although characteristic pathological findings of neurofibroma were present in some biopsy specimens in this case, it still took about 3 years before a pathological diagnosis was made. Therefore, it is important for endoscopists to ask pathologists to stain biopsy specimens for S‐100 when neurofibroma is suspected.

## CONFLICT OF INTEREST

The authors declare no conflict of interest.

## FUNDING INFORMATION

The authors declare no funding information.
